# In vitro anticancer, antioxidant and chelating activities of natural organosulfur compounds originated from Türkiye: an investigation on breast and colorectal cancer cells

**DOI:** 10.55730/1300-0144.5970

**Published:** 2025-01-26

**Authors:** Ümmügülsüm POLAT KORKUNÇ, Hilal ÇALIK, Leyla POLAT KÖSE, Rabia ÇAKIR KOÇ, Emine KARAKUŞ

**Affiliations:** 1 Department of Chemistry, Faculty of Arts and Science, Yıldız Technical University, İstanbul, Turkiye; 2 Department of Bioengineering, Faculty of Chemical and Metallurgical Engineering, Yıldız Technical University, İstanbul, Turkiye; 3 Health Institutes of Turkey (TUSEB), Turkey Biotechnology Institute, İstanbul, Turkiye; 4 Department of Pharmacy Services, Vocational School, İstanbul Beykent University, İstanbul, Turkiye; 5 Health Biotechnology Joint Research and Application Center of Excellence, İstanbul, Turkiye

**Keywords:** Kastamonu Taşköprü garlic, organosulfur compounds, cell viability, breast cancer, colorectal cancer, antioxidant activity, metal chelating capacity

## Abstract

**Background/aim:**

Taşköprü garlic, cultivated in the Taşköprü region of Kastamonu Province, is highly renowned in Türkiye. This study aimed to determine the anticancer and antioxidant effects of extracts from Kastamonu Taşköprü garlic on human breast cancer and colorectal cancer cells.

**Materials and methods:**

Taşköprü garlic, which contains natural organosulfur compounds (OSCs), has a geographical registration. Garlic contains oil- and water-soluble OSCs known to exhibit anticancer activity by interfering with MCF-7 and Caco-2 proliferation and tumor metastasis. This study assessed the antiproliferative activity of oil- and water-soluble garlic extracts with and without glutathione at different concentrations using the XTT assay on NIH/3T3, MCF-7, and Caco-2 cell lines over 24 h. In addition, the reducing capacity, radical scavenging activity, and metal chelation activity of OSCs in Taşköprü garlic were analyzed.

**Results:**

Both oil- and water-soluble garlic extracts significantly inhibited the proliferation of MCF-7 and Caco-2 in a dose-dependent manner after 24 h of incubation. The half-maximal inhibition concentration (IC_50_) values of OSCs and positive controls for N,N-dimethyl-p-phenylenediamine cation (DMPD^.+^) radical removal and 2,2′-bipyridyl-Fe^2+^ chelation activity were 129.593–1004.346 and 165.065–495.195 μg/mL, respectively. Furthermore, the reducing impact of OSCs and positive controls were evaluated based on their cupric ion (Cu^2+^) reducing capabilities. All results were compared with the respective positive controls.

**Conclusion:**

The findings revealed that oil-soluble garlic extracts exhibited anticancer properties against both Caco-2 and MCF-7 cancer cells, without inducing any cytotoxicity in non-cancerous NIH/3T3. In addition, water-soluble extracts have higher antiproliferative activity on Caco-2 and MCF-7 cells in a dose-dependent manner compared to oil-soluble extracts. However, they also exhibited notable cytotoxicity on fibroblast cells. OSCs showed limited activity in reduction and radical scavenging assays but demonstrated strong chelating activity. These results suggest that both water-soluble and oil-soluble garlic extracts hold promising anticancer potential against both MCF-7 and Caco-2.

## Graphical Abstract

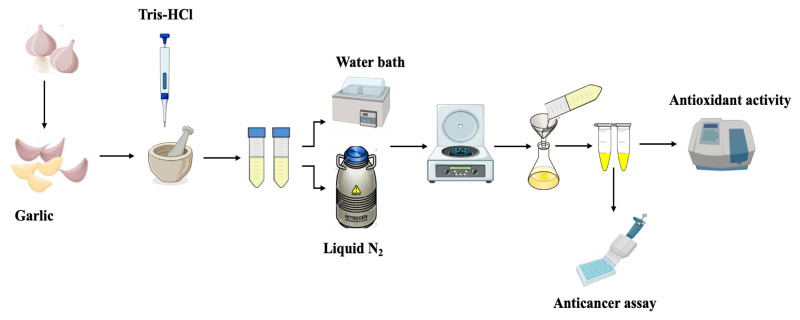

## 1. Introduction

Organosulfur compounds (OSCs) are phytochemicals found as secondary metabolites in many species of the genus *Allium*. This genus encompasses about 600 species, primarily consisting of garlic, onions, and leeks [1]. Natural OSCs have a wide range of biological activities. Because of its sulfur content, garlic is one of the most significant sources of organosulfur compounds [2]. These sulfur compounds are responsible for the distinctive flavor and odor of garlic and garlic-containing supplements [3]. The structures of 33 OSCs have been identified, and the biological activities of a number of them are known. The average total OSC concentration in 1 g of raw garlic ranges from 11 to 35 mg, which is nearly four times higher than the concentrations found in other cruciferous vegetables, such as onions and broccoli [4]. The pungent aroma of garlic is attributed to its sulfur-containing chemicals, including allicin, alliin, diallyl sulfide, and dithiin [5]. OSCs are categorized into two groups: oil-soluble and water-soluble OSCs [6]. Both types of OSCs found in garlic have demonstrated significant efficacy in preventing cancer. The oil-soluble OSCs found in garlic are highly effective in the initial phase of cancer development, while the water-soluble OSCs in garlic are regarded as the primary active component for treating cancer [7]. OSCs boost the activity of enzymes involved in cancer cell detoxification [8]. Garlic’s antiproliferative properties also play a role in the mechanism by which cancer cells undergo apoptosis. Key OSCs, including DAS, DADS, DATS, and ajoene, have been shown to promote cancer cell apoptosis by increasing DNA fragmentation and intracellular free calcium, upregulating p53 and Bax, and downregulating Bcl-2. Additionally, studies have shown that garlic enhances the immune response, thereby reducing the risk of malignancy [6].

Colorectal cancer (CRC) is the third most prevalent cancer, accounting for over 1.9 million cases annually, which represents 10% of all new cancer diagnoses worldwide [9]. Researchers have found that OSCs and their derivatives can help fight CRC by stopping cell growth and causing apoptosis [10]. In a study investigating the cytotoxic effect of organosulfur compounds on primary CRC cells, DATS was found to trigger apoptosis in human CRC cells via the mitochondria-dependent apoptosis pathway [11].

Breast cancer (BC) rates are the second main cause of mortality among women globally [12]. Breast cancer is the main cause of cancer-related mortality and the most commonly diagnosed cancer among women in 140 of the 184 nations worldwide [13]. Water-soluble OSCs inhibited the growth, migration, invasion, metastasis, and colony-forming abilities of BC cells. A study also investigated how oil-soluble garlic compounds like allicin might help fight breast cancer. Allicin stopped MCF-7 cells from multiplying and slowed down the cell cycle by increasing the number of cells in the G0/G1 and G2/M phases and decreasing the number of cells in the S phase [7].

Türkiye is one of the leading garlic producers worldwide [14]. Kastamonu Taşköprü garlic is classified as a Geographical Indication food product. The agricultural soils of Taşköprü District are rich in selenium, leading to high concentrations of selenium, resulting in high levels of selenium and organosulfur compounds that have anticancer and cancer risk-reducing properties. A comparison between Kastamonu Taşköprü garlic and Chinese garlic revealed that Taşköprü garlic contained higher levels of DADS, a compound with anticancer properties, whereas DATS was not detected in Chinese garlic [15]. Most other garlic varieties contain little to no selenium and OSCs, which are known for their anticancer properties and potential to reduce cancer risk [16,17]. The high dry matter concentration of Taşköprü garlic enables long-term preservation without degradation. Methyl jasmonate, a methyl ester of jasmonic acid, is thought to be efficacious in combating illnesses and extending the storage life of garlic. Selenium increases the biosynthesis and transmission of jasmonic acid, which keeps its high antioxidant content. This is why jasmonic acid is linked to selenium tolerance [18,19].

Glutathione (GSH) is produced by all organs and cells [20]. Studies have shown that cancer cells contain approximately four times higher GSH concentrations than normal cells, and GSH plays a critical role in cancer cell survival [21]. Recent studies have preferred glutathione disulfide conjugates due to their ease of synthesis and biocompatibility [22].

Both in vitro and in vivo models are crucial tools for understanding human disease, each with their own advantages and disadvantages. In vitro investigations offer cost-effective reagents and exceptional repeatability compared to in vivo experiments [23].

In living systems, antioxidants are natural mechanisms that ensure the healthy continuation of metabolic cycles. Their primary function is to counteract radical threats that may arise within the organism or from external sources [24,25]. The body maintains a balance between antioxidant systems and free radicals. When this balance favors the antioxidant systems, the organism can sustain its vital functions in a healthy state. However, when this equilibrium shifts in favor of the radicals, a condition known as oxidative stress occurs [26,27]. In such cases, the demand for natural antioxidant sources increases. Biological processes such as oxygen respiration, immune responses, fat oxidation, metabolism, and infections contribute to free radical formation [28]. In addition, various factors such as stress, smoking, air pollution, alcohol consumption, poor dietary habits, and exposure to heavy and toxic metals can lead to free radical formation in the body [24,29]. Measures can be taken to mitigate or prevent their harmful effects on the body. The body’s natural antioxidant systems help control free radicals in response to radical damage. These systems complement each other, as they are localized in different cells and target various types of free radicals [30]. By neutralizing free radicals, antioxidants themselves undergo oxidation, thereby preventing further oxidative damage [28]. As a result, antioxidant-rich foods are essential for maintaining health [28,29]. Accordingly, research on the antioxidant capacity of plant-based resources is steadily increasing [31,32]. The data obtained from such studies are expected to aid in the design, synthesis, and pharmacological applications of therapeutic drugs. Additionally, increasing restrictions on the use of synthetic antioxidants have heightened interest in natural alternatives, driving research efforts to discover new antioxidant sources [26,32].

*Allium* species contain numerous therapeutically and chemopreventively important constituents, including flavonoids and OSCs that regulate cellular and molecular mechanisms. This study is unique because, although research exists on allyl sulfur compounds related to prevalent cancer types like BC and CRC, there is a lack of studies that focus on Taşköprü garlic, which is widely consumed and exported worldwide. No previous studies on OSCs in the oil- and water-soluble components of Taşköprü garlic have been determined. Additionally, in this study, we aimed to investigate how the allyl sulfur compounds DAS, DADS, and DATS function as antioxidants, radical scavengers, and metal chelators.

## 2. Materials and methods

### 2.1. Reagents

Garlic is harvested from Taşköprü District of Kastamonu Province. Glutathione and acetonitrile were purchased from Sigma Aldrich and Merck, respectively. NIH/3T3 mouse embryo fibroblast cell line (ATCC CRL-1658), Caco-2 human colorectal cancer cell line (ATCC HTB- 37), and MCF-7 human breast adenocarcinoma (ATCC HTB-22) were purchased from American Type Culture Collection (ATCC). Dulbecco’s modified Eagle’s medium/Nutrient Mixture Ham’s F-12 (DMEM/F-12), fetal bovine serum (FBS), penicillin-streptomycin solution, phenazine methosulfate (PMS) and trypsin/EDTA (0.25%) were obtained from Sigma-Aldrich (St Louis, MO, USA). 2,3-bis (2-methoxy-4-nitro-5-sulfophenyl)-2H-tetrazolium-5-carboxanilide (XTT) was purchased from Biomatik (USA). Butylated hydroxyanisole (BHA), butylated hydroxytoluene (BHT), trolox, and *α*-tocopherol, N,N-dimethyl-*p*-phenylenediamine (DMPD), CuCl_2_, 2,2′-Bipyridyl were obtained from Sigma-Aldrich GmbH (Sternheim, Germany).

### 2.2. Kastamonu Taşköprü garlic extraction method

**Figure 1:**
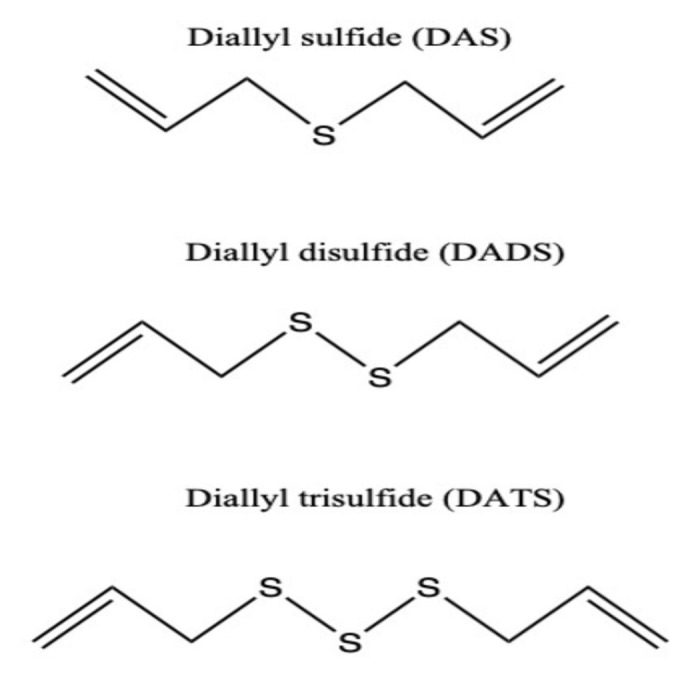
Molecular structure of the main natural organosulfur compounds present in Taşköprü garlic.

Garlic samples were harvested from Taşköprü District of Kastamonu Province. The target organosulfur compounds (DAS, DADS, and DATS) were analyzed in oil-soluble and water-soluble extractions of garlic [33]. The molecular structures of these compounds are given in Figure 1 [33,34].

Garlic bulbs from Taşköprü were peeled and cleaned using distilled water. Since there are water-soluble and oil-soluble compounds in the garlic content, two different extraction methods were applied to the same garlic samples [6]. First, water-soluble garlic extractions were prepared, and then oil-soluble garlic extractions were prepared.

When garlic samples are crushed with a mortar and pestle, alliin molecules are converted to allicin using the enzyme alliinase. Allicin molecule rapidly forms organosulfur compounds (DAS, DADS, and DATS) [35].

In order to ensure conjugation of glutathione in the intracellular fluid with allyl sulfur compounds, water-soluble cold and hot extractions of garlic were carried out by crushing approximately 5.0 g of Taşköprü garlic cloves in the presence of 100 mM (200 μL) GSH (GCW and GHW) in 10 mL of 50 mM Tris-HCl buffer (pH 7.5). Water-soluble glutathione-free cold and hot (CW and HW) extractions of garlic samples were then crushed in 10 mL of 50 mM Tris-HCl buffer (pH 7.5). Crushing was performed at room temperature for approximately 10 min, with extractions performed at both low and high temperatures. For cold extraction (CW and GCW), the crushed garlic samples were stored in liquid nitrogen (N_2_). For hot extraction (HW and GHW), the crushed garlic was heated in a 100 °C water bath. All prepared water-soluble garlic extracts were centrifuged at +4 °C and 9000 rpm for 45 min.

After centrifugation, all water-soluble garlic extracts (CW, HW, GCW, and GHW) were obtained using a 0.45-mm filter. The water-soluble garlic extracts were stored at −20 °C until antioxidant and anticancer studies were performed. The remaining pellets from the water-soluble garlic extracts were incubated in 10 mL of acetonitrile for 24 h at room temperature to obtain oil-soluble garlic extracts.

After incubation, the obtained oil-soluble extracts (HO, CO, GHO, and GCO) were centrifuged at 9000 rpm for 45 min at +4 °C. Oil-soluble extracts were then filtered using a 0.45-mm filter, and the oil-soluble garlic extracts were stored at −20 °C until further analysis.

### 2.3. Cell culture studies

NIH/3T3, Caco-2, and MCF-7 cells were cultured at 37 °C in a 5% CO_2_ incubator in DMEM-F12 medium supplemented with 10% FBS and 1% penicillin-streptomycin solution. When cells reached confluency, they were detached with trypsin/EDTA solution and centrifuged at 1000 rpm for 5 min. The cells were resuspended in the culture medium and used for further experiments.

### 2.4. Antioxidant assays

The antioxidant, antiradical, and chelating properties of allyl sulfur compounds in Taşköprü garlic were investigated. The cupric reducing antioxidant capacity (CUPRAC), DMPD^·+^ cation radical scavenging, and bipyridyl-metal chelation methods were employed for this purpose. To assess the reliability of our research and explore the behavior of allyl sulfur compounds in Taşköprü garlic under varying reaction conditions, we employed multiple methodologies.

#### Cu^2+^-Cu^+^ reduction capacity (CUPRAC method)

In order to determine the reducing capacity of allyl sulfur compounds, the Cu^2+^-Cu^+^ reduction method (CUPRAC) developed by Apak et al. [36] was used with slight modifications. First, positive standards and allyl sulfur compounds (10–30 μg/mL) were pipetted into test tubes at varying concentrations. Following this, CuCl_2_ solution (10 mM, 0.125 mL), neocuproine solution (7.5 (×)10^−3^ M, 0.125 mL), and pH 6.5 acetate buffer (1 M, 0.125 mL) were added, respectively. The final volume was completed to 1 mL with deionized water, and the mixture was shaken vigorously. After incubation, absorbance values at 450 nm were measured using a spectrophotometer (PerkinElmer Lambda 25 UV/Vis, USA).

#### DMPD^·+^ cation radical scavenging capacity method

For the N,N-dimethyl-p-phenylenediamine cation (DMPD^·+^) radical scavenging activity study, the method developed by Fogliano et al. [37] was used. As per the method’s principle, we prepared all solutions fresh. First, a 100 mM solution of DMPD in water was prepared. One milliliter of this solution was added to 100 mL of 0.1 M acetate buffer with a pH of 5.25. Following this, 0.2 mL of a 0.05 M FeCl_3_ solution was added to this mixture, and DMPD^·+^ cation radical was obtained. Next, allyl sulfur compounds (10–30 μg/mL) were pipetted into test tubes at varying concentrations. One milliliter of DMPD^·+^ cation radical solution was added, and the final volume was adjusted to 1.5 mL with deionized water. After incubation, spectrophotometric measurements were made at 505 nm, and the values were recorded.

#### 2,2′-Bipyridyl-Fe^2+^ chelating method

The Ferrous (Fe^2+^) chelating property with allyl sulfur compounds in geographically registered Taşkörü garlic was determined [38,39]. For this purpose, all solutions were first prepared fresh. Next, certain concentrations (10–30 μg/mL) of all three allyl sulfide compounds were put into test tubes. Following this, 0.125 mL of FeSO_4_ solution (1 mM) and 0.500 mL of Tris-HCl buffer solution with a pH of 7.4 were added, respectively, and vortexed vigorously. Afterwards, 0.500 mL of 0.1% 2,2′-bipyridyl solution (in 0.2 M HCl) and 1.250 mL of ethanol were added. The final volume was completed to 3 mL with distilled water, and the mixture was vortexed. The test tubes were incubated in the dark at room temperature, and then their absorbance at 522 nm was measured and recorded. To obtain the standard graph, EDTA was used.

The percentage of DMPD^·+^ cation radical and Fe^2+^ chelating removal was computed using the following equation:


SE (%)=[1-(As/Ac)] (×)100,

where SE is scavenging effects, A_C_ is the absorbance value of the control, and A_S_ is the absorbance value of the sample [40].

### 2.5. Cell viability assay

The cytotoxic effects of Taşköprü garlic extracts were assessed using XTT cell viability assay according to ISO 10993-5-2009 standard. Briefly, NIH/3T3, Caco-2, and MCF-7 cells were initially seeded onto 96-well tissue culture plates at a density of 1(x)10^4^ cells/well and were incubated at 37 °C with 5% CO_2_ for 24 h. The monolayered cells were exposed to various concentrations (5, 10, 25, 50, and 100 mg/mL) of the Taşköprü garlic extracts and further incubated at 37 °C for 24 h. Untreated cells were used as the negative control. Following the incubation period, the cell culture medium was replaced with fresh DMEM/F-12 medium containing 0.4 mg/mL XTT powder and 0.1% PMS. The cells were then incubated for 4 h at 37 °C in 5% CO_2_ and the absorbance values were measured at 450 nm using an ELISA microplate reader (Lab-Line Inc., Odessa, TX, USA). Finally, the percent cell viability was calculated according to the equation below:


$$\text{Cell Viability }(\%)=({\text{OD}}_{450}\;\text{of extracts}/{\text{OD}}_{450}\;\text{of control})\times 100.$$

### 2.6. Statistical analysis

Antioxidant assay studies were carried out in triplicate. The obtained data were analyzed using SPSS (version 11.5 for Windows 2000, SPSS Inc.), and the mean and standard deviation were reported. One-way analysis of variance (ANOVA) was applied. Significant differences between means were determined using Duncan’s multiple range test, with p < 0.05 considered significant and p < 0.01 considered highly significant. Statistical analysis of the cell viability assay was performed using GraphPad Prism software with an unpaired Student’s t-test. All data are presented as the mean and standard deviation. Each group was tested in triplicate. Differences were considered statistically significant at p < 0.05 and p < 0.001.

## 3. Results

### 3.1. Effects of the Kastamonu Taşköprü garlic extract on the fibroblast cell viability

**Figure 2:**
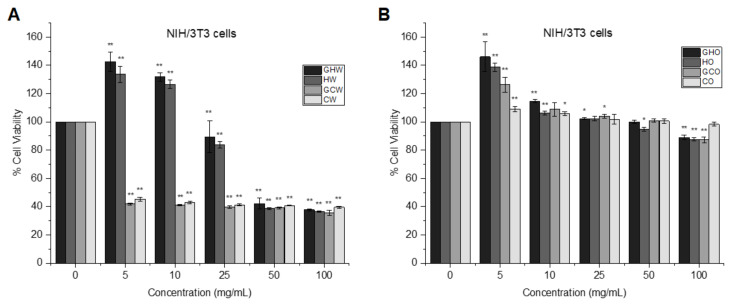
NIH/3T3 cell proliferation after 24 h of treatment with (a) water-soluble garlic extracts and (b) oil-soluble garlic extracts. *p < 0.05 and **p < 0.001 vs. control.

The effects of Taşköprü garlic extracts on the growth of noncancerous NIH/3T3 mouse fibroblast cells were investigated using the XTT assay to evaluate the potential toxic side effects of garlic extracts and further determine the optimal concentrations. As shown in Figure 2A, both CW and GCW extracts at all concentrations decreased the viability of NIH/3T3 cells to approximately 35%–45% compared to the control (p < 0.001). After 24 h of exposure, about 65% and 60% of NIH/3T3 cells were viable at concentrations of 50 mg/mL and 100 mg/mL for HW and GHW extracts (p < 0.001). In Figure 2B, the NIH/3T3 cell viability was about 87%–100% following exposure to HO, CO, GHO, and GCO extracts. This indicated that water-soluble garlic extracts showed dose-dependent cytotoxic effects, while oil-soluble extracts did not show considerable cytotoxic effects on fibroblast cells.

### 3.2. Effects of the Kastamonu Taşköprü garlic extract on the cancer cells

**Figure 3:**
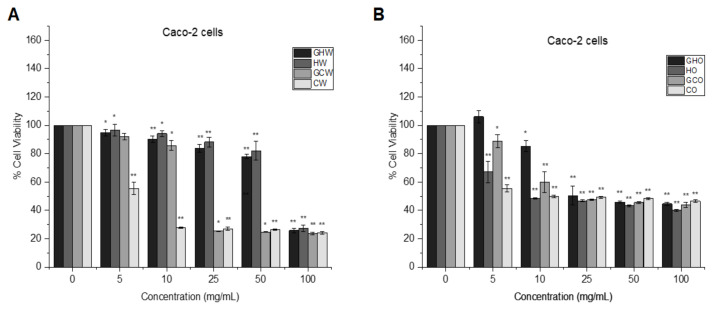
Caco-2 cell proliferation after 24 h of treatment with (a) water-soluble garlic extracts and (b) oil-soluble garlic extracts. *p < 0.05 and **p < 0.001 vs. control.

The antiproliferative effects of garlic extracts on Caco-2 colorectal cancer and MCF-7 breast cancer cells were determined using the XTT assay. As indicated in Figure 3A, HW and GHW extracts had significant cytotoxic effects on Caco-2 cells at 100 mg/mL concentration by decreasing the cell viability to around 23%–27% (p < 0.001). Similarly, CW and GCW extracts at 25 mg/mL, 50 mg/mL, and 100 mg/mL reduced the Caco-2 cell viability to around 24%–27% (p < 0.001). In Figure 3B, GHO and GCO extracts demonstrated a reduction in the Caco-2 cell viability to about 43%–55% at the doses of 25 mg/mL, 50 mg/mL, and 100 mg/mL (p < 0.001), while HO and CO extracts exhibited considerable antiproliferative effects at all the concentrations, reducing cell viability to around 40%–45%.

**Figure 4:**
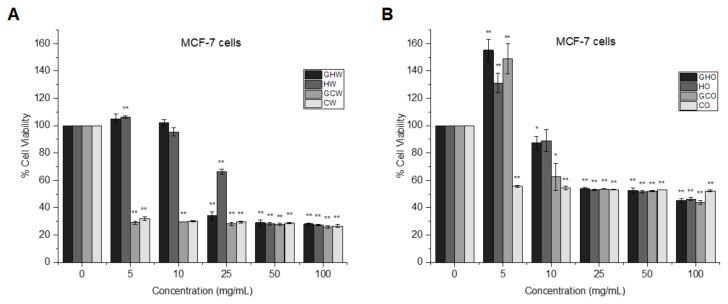
MCF-7 cell proliferation after 24 h of treatment with (a) water-soluble garlic extracts and (b) oil-soluble garlic extracts. *p < 0.05 and **p < 0.001 vs. control.

As shown in Figure 4A, the water-soluble cold garlic extracts (GCW and CW) showed a significant inhibition of MCF-7 cell proliferation at all concentrations, whereas the water-soluble hot garlic extracts (GHW and HW) exhibited a dose-dependent antiproliferative activity, decreasing the cell viability as low as 28% at 50 mg/mL and 100 mg/mL concentrations (p < 0.001). In Figure 4B, all oil-soluble garlic extracts at the doses of at 25, 50, and 100 mg/mL showed a moderate antiproliferative effect on MCF-7 by reducing the cell viability to approximately 50% (p < 0.001).

Consequently, oil-soluble garlic extracts at doses of 25, 50, and 100 mg/mL exhibited anticancer properties against both Caco-2 and MCF-7 cancer cells, without inducing any cytotoxicity in noncancerous fibroblast cells after 24 h of incubation. In addition, water-soluble extracts have higher anticancer activity on Caco-2 and MCF-7 cells in a dose-dependent manner compared to oil-soluble extracts. However, their significant cytotoxicity on fibroblast cells suggests that they may not be appropriate for chemotherapeutic purposes. The potential anticancer properties of garlic extracts against colorectal and breast cancer could be attributed to the various oil-soluble and water-soluble organosulfur compounds present in the extracts [7,41]. In addition, it has been observed that glutathione content in garlic extracts did not show a statistically significant antiproliferative impact on both cancerous and fibroblast cells. Nevertheless, their significant cytotoxicity on fibroblast cells suggests that they may not be appropriate for pharmaceutical use.

### 3.3. Antioxidant assays

**Figure 5:**
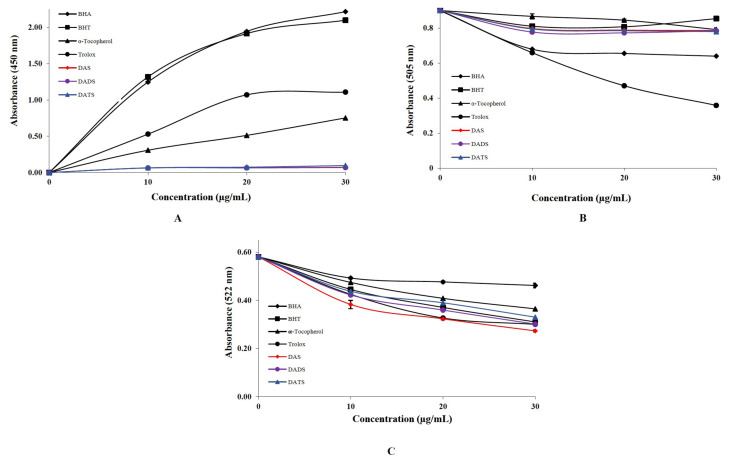
**A)** Comparison of cupric ion (Cu^2+^) reducing abilities of OSC and reference antioxidants at different concentrations (10–30 mg/mL). **B)** Comparison of the DMPD^·+^ radical scavenging ability of OSC and reference antioxidants at different concentrations (10–30 mg/mL). **C)** Comparison of the 2,2′-Bipyridyl-Fe^2+^ chelating effect of OSC and reference antioxidants at different concentrations (10–30 mg/mL). [butylated hydroxyanisole (BHA), butylated hydroxytoluene (BHT); trolox; *α*-tocopherol; Diallyl sulfide (DAS); Diallyl disulfide (DADS); Diallyl trisulfide (DATS).

Within the scope of the study, the antioxidant, antiradical, and chelating capacities of allyl sulfide compounds in geographically registered Taşköprü garlic were also proven. Thus, the activities of allyl sulfide compounds were determined, and the biological activities of each compound were compared. The obtained results were remarkable. The Cu^2+^-reducing capacities of the DAS, DADS, and DATS and standards are shown in Figure 5. The Cu^2+^ reducing capacities of the DAS, DADS, DATS, and positive controls an equal concentration (30 μg/mL) were as follows: BHA (2.216; r^2^: 0.8885) > BHT (2.099; r^2^: 0.8320) > Trolox (1.108; r^2^: 0.8937) > *α*-tocopherol (0.751; r^2^: 0.9906) > DATS (0.096; r^2^: 0.8186) > DAS (0.071; r^2^: 0.5666) > DADS (0.068; r^2^: 0.5324). In the Cu^2+^ reducing method, BHA, a standard antioxidant molecule, exhibited the strongest reducing power, while DADS exhibited the weakest reducing power. Additionally, all three allyl sulfide compounds demonstrated lower activity compared to the positive controls. Because it is practical, selective, straightforward, quick, and inexpensive, the CUPRAC method is significant for many different kinds of compounds or herbal sources that have antioxidant characteristics.

The radical scavenging ability was assessed by investigating the capability to remove DMPD^·+^ cation radicals. As higher quantities of antioxidants were introduced to the radical solutions, the dark pink hue of the reaction medium became paler. Table presents the statistically significant decrease (p < 0.01) in the concentration of DMPD^·+^ cation radicals due to the enhanced radical scavenging capabilities of DAS, DADS, DATS, BHA, BHT, Trolox, and *α*-tocopherol. The DMPD^·+^ cation radical scavenging activity and IC_50_ values are as follows: Trolox (129.593 μg/mL; r^2^: 0.9983) > BHA (286.956 μg/mL; r^2^: 0.6357) > *α*-tocopherol (1004.346 μg/mL; r^2^: 0.9597) > DAS (669.564 μg/mL; r^2^: 0.5471) = DADS (669.564 μg/mL; r^2^: 0.3525) = DATS (669.564 μg/mL; r^2^: 0.5833). The low IC_50_ values obtained from the activity test conclusively demonstrate that the antioxidant compounds exhibit significant DMPD^·+^ cation radical scavenging effect. The absorbance results for this technique were obtained at a wavelength of 505 nm [37]. Allyl sulfide compounds did not show excellent properties in Cu^2+^ reduction and DMPD^·+^ cation radical scavenging tests. However, the metal chelating activity of 2,2′-bipyridyl was significantly strong. The order of the 2,2′-bipyridyl metal chelating activity is illustrated in Figure 5, and IC_50_ values were as follows: DAS (165.065 μg mL; r^2^: 0.9165) > Trolox (178.270 μg/mL; r^2^: 0.9340) > DADS (193.772 μg/mL; r^2^: 0.9606) > BHT (202.580 μg/mL; r^2^: 0.9855) > DADS (222.838 μg/mL; r^2^: 0.9526) > *α*-tocopherol (278.547 μg/mL; r^2^: 0.9778) > BHA (495.195 μg/mL; r^2^: 0.7589). All three allyl sulfide compounds exhibited effective chelating properties. As shown in Table, the results were quite close to those of the positive standard, trolox. Among the positive controls, trolox showed the best chelating activity. However, DAS’s chelating activity was even better than trolox’s. The fact that DAS showed better chelating activity than the other two allyl sulfide compounds is due to the fact that it contains two electron-donating groups adjacent to the sulfur atom. There are disulfide bonds between the sulfur atoms in DADS and DATS compounds. Therefore, there is one electron-donating group in these compounds. Consequently, as the electron density on the sulfur atom increases, the chelating activity also increases.

## 4. Discussion

Anticancer properties of water-soluble and oil-soluble garlic extracts were examined using XTT cell viability assay. As a result, oil-soluble garlic extracts at doses of 25, 50, and 100 mg/mL exhibited anticancer properties against both Caco-2 and MCF-7 cancer cells, without inducing any cytotoxicity in noncancerous fibroblast cells after 24 h of incubation. Besides, water-soluble extracts have higher anticancer activity on Caco-2 and MCF-7 cells in a dose-dependent manner compared to oil-soluble extracts. In addition, it was observed that glutathione content in garlic extracts did not show a statistically significant antiproliferative impact on either cancerous or fibroblast cells. The potential anticancer properties of garlic extracts against colorectal and breast cancer could be attributed to the various oil-soluble and water-soluble organosulfur compounds present in the extracts [7,35].

However, water-soluble garlic extracts exhibited significant cytotoxicity on fibroblast cells when compared to oil-soluble extracts. Water-soluble garlic extracts, containing highly reactive compounds like SAC, SAMC, and S-methyl cysteine, can easily penetrate cell membranes and disrupt biological processes in both cancerous and healthy cells, leading to cytotoxicity [42]. In contrast, oil-soluble extracts, which include lipophilic compounds such as DAS, DADS, and DATS, may be more selective, primarily targeting cancer cells while sparing healthy cells [28]. Their anticancer mechanisms include inducing apoptosis, detoxifying carcinogens, reducing oxidative stress, preventing DNA adducts, and regulating the cell cycle [43]. Due to structural and metabolic differences between cancerous and healthy cells, oil-soluble compounds may achieve greater selectivity. Additionally, studies suggest that oil-soluble compounds are more effective in cancer prevention than water-soluble ones [44]. Therefore, oil-soluble extracts may serve as more suitable agents for anticancer therapies due to their specific effects on cancer cells while preserving fibroblasts.

Positive controls in studies commonly include BHA, BHT, trolox, and *α*-tocopherol, all known for their strong antioxidant properties [45,46]. Their significance and the reasons for their selection are explained in detail.

Both BHA and BHT are phenolic compounds effective at reducing free radicals and preventing lipid degradation [45]. They are lipid-soluble and stable across a range of temperatures, making them suitable for use in food, cosmetics, and pharmaceuticals [45]. BHA is commonly used as a reference compound in studies assessing antioxidant properties, particularly in lipophilic systems [45]. Moreover, BHT’s high thermal stability makes it suitable for experiments conducted at elevated temperatures [47]. α-Tocopherol, a naturally occurring antioxidant, efficiently inhibits lipid peroxidation in biological systems [46]. As a natural antioxidant, it is highly biocompatible and widely regarded as a reliable reference under physiological conditions [46]. Trolox, a vitamin E derivative, scavenges free radicals in a manner similar to α-tocopherol but is also water-soluble [48]. Its solubility in water makes it ideal for aqueous systems and cell culture experiments.

These compounds have well-defined antioxidant properties, making them valuable for evaluating the effectiveness of novel or natural antioxidants [45]. They exhibit strong and consistent radical-scavenging abilities, often outperforming other antioxidants in terms of stability and activity [47]. Their widespread use in the literature facilitates cross-study comparisons, ensuring the consistency of experimental results. These compounds are selected based on the type of research (lipophilic or aqueous environment), the radical source, and the experimental conditions, providing a robust framework for assessing antioxidant activity [47].

## 5. Conclusion

The Taşköprü garlic extraction method revealed the natural organosulfur compounds DAS, DADS, and DATS through an oil-soluble extraction procedure [33]. Recently, there has been growing research interest in garlic for cancer treatment. GSH-dependent enzymes, particularly GSH S-transferase, catalyze the majority of nonenzymatic GSH conjugation reactions. Through these reactions, GSH can conjugate with anticancer drugs or their byproducts, forming compounds that are less toxic or easier to eliminate. On one hand, this process reduces the likelihood of anticancer drugs damaging healthy, non-cancerous cells. On the other hand, it may reduce the effectiveness of anticancer drugs on cancerous cells, potentially leading to early development of drug resistance [49]. The majority of malignant cells have a higher ROS set point than their associated noncancerous cells. In numerous types of tumor cells, these elevated levels facilitate their proliferation, growth, metastasis, and survival in a variety of microenvironments or conditions.

Research has shown that exogenously added GSH inhibits the inflammatory response by regulating ROS. Breast, lung, and ovarian cancers, as well as cancers of the head and neck, have been linked to elevated levels of GSH [50].

In anticancer studies, the findings about glutathione in cancer cells are contradictory. Some researchers have found GSH levels to be high for cancer cells, while others have observed constant or low levels [51–53].

The cell viability results exhibited that water-soluble and oil-soluble garlic extracts exhibit promising anticancer potential against both colorectal and breast cancers after 24-hour exposure, in a dose-dependent matter. This study yielded significant pharmacological results regarding garlic, particularly Taşköprü garlic, when applied to these cancer cell lines. This study anticipates that it will aid in the treatment of these cancer types with synthetic or semisynthetic drugs. Further analysis is necessary to investigate the progression of OSCs into effective cancer treatments.

It has been proven by many studies that Türkiye is a region rich in biodiversity. Many of the natural resources grown in the region are used to prevent diseases or support treatment. Therefore, the biological activities of such natural resources need to be proven. In this study, the antioxidant, antiradical, and metal chelating activities of allyl sulfide compounds in geographically registered Taşköprü garlic were examined. In reduction and radical removal studies, positive controls showed better activity than allyl sulfide compounds. However, allyl sulfide compounds showed remarkably strong chelating effects. Allyl sulfide compounds showed excellent metal chelating activity thanks to the increased electron density on the sulfur atom. With the development of industrialization, the rate of chemical waste in air, water, and soil continues to increase. Accordingly, heavy and toxic metals can enter the human body even from the air we breathe. For this reason, we anticipate that OSCs, found in Taşköprü garlic and responsible for its characteristic taste and odor, will help minimize heavy metal accumulation, especially when consumed from natural sources. In fact, we think that regular daily consumption of this natural source will prevent metal accumulation or the negative reactions that may be caused by them, thus protecting against cancer.

It is important to learn more about how garlic-derived allyl sulfides work as medicines because not only does that help us understand their effects, but it also makes them more useful in clinical settings.

## Figures and Tables

**Table:** Comparison of the reducing power of allyl sulfide compounds at the same concentration (30 μg/mL) based on Cu^2+^ reducing capacity using the CUPRAC method, as well as the IC_50_ values determined for in DMPD^·+^ cation radical and 2,2′-bipyridyl- metal chelating assays. [butylated hydroxyanisole (BHA), butylated hydroxytoluene (BHT); trolox; α-tocopherol; Diallyl sulfide (DAS); Diallyl disulfide (DADS); Diallyl trisulfide (DATS).

Compounds	Cu^2+^- Cu^+^ reducing		DMPD^·+^ scavenging		2,2′-bipyridyl metal chelating	
	λ_450_*	r^2^	IC_50_ (μg/mL)	r^2^	IC_50_ (μg/mL)	r^2^
**BHA**	2.216±0.003	0.8885	286.956	0.6357	495.195	0.7589
**BHT**	2.099±0.005	0.8320	-	-	202.580	0.9855
***a*** **-Tocopherol**	0.751±0.006	0.9906	1004.346	0.9597	278.547	0.9778
**Trolox**	1.108±0.004	0.8937	129.593	0.9983	178.270	0.9340
**DAS**	0.071±0.002	0.5666	669.564	0.5471	165.065	0.9165
**DADS**	0.068±0.003	0.5324	669.564	0.3525	193.772	0.9606
**DATS**	0.096±0.010	0.8186	669.564	0.5833	222.838	0.9526

* expressed as absorbance values.
